# Advancing Cardiovascular Disease Prevention in Asian Populations

**DOI:** 10.1016/j.jacasi.2026.07.010

**Published:** 2026-08-18

**Authors:** Nathan D. Wong, Dong Zhao

**Figure:**
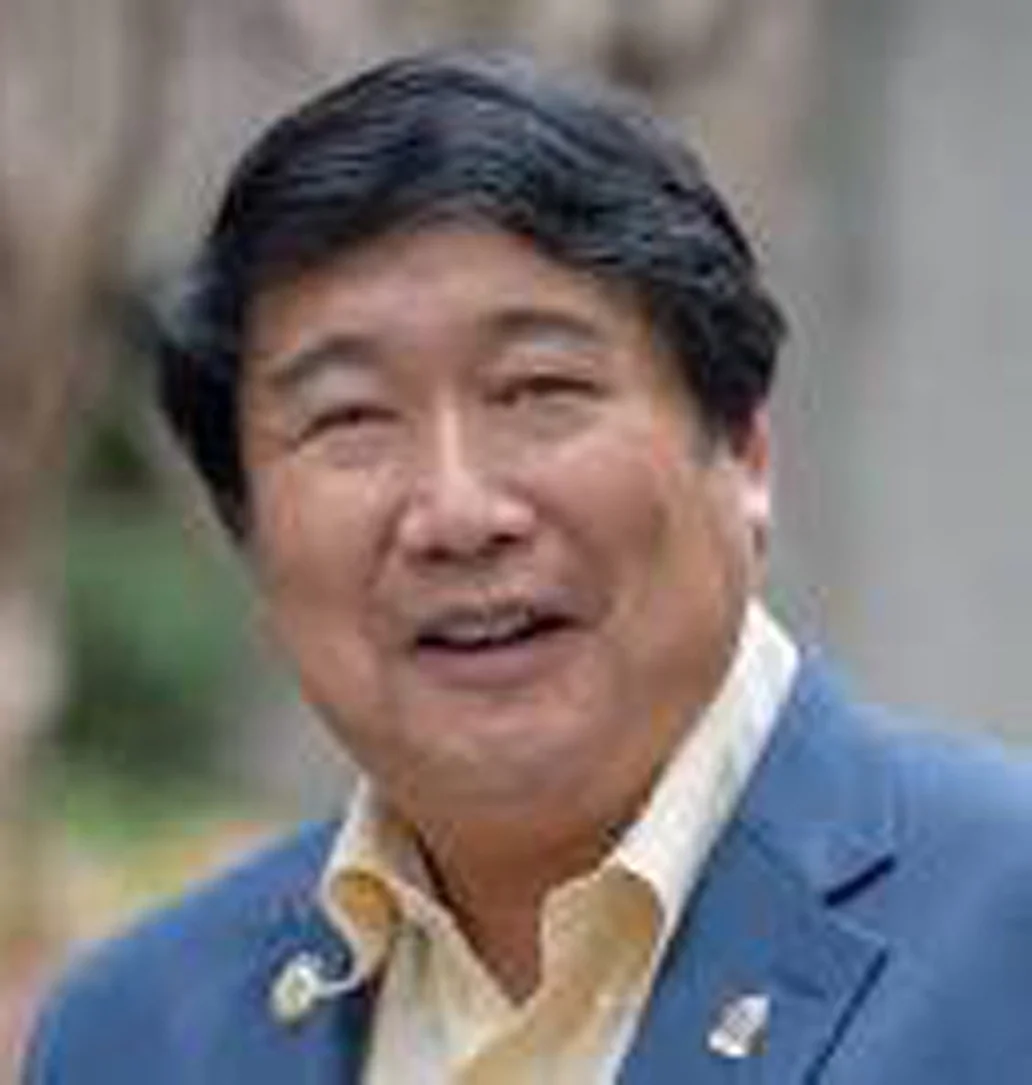

**Figure:**
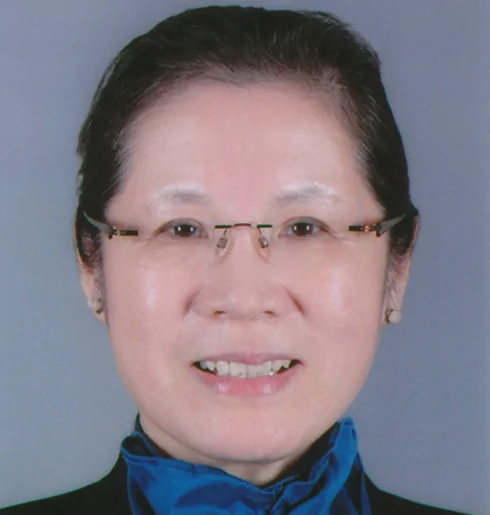

With cardiovascular diseases (CVDs) remaining the leading causes of death globally and within Asia, there remains a continuing need to improve efforts at primordial, primary, and secondary prevention. Despite guidelines and recommendations promoting CVD prevention efforts among Asian populations throughout Asia and beyond,^1^^,^^2^ resources devoted to such efforts remain limited locally, nationally, and regionally and comprise a small proportion of health care dollars spent. With the rapid development many parts of Asia have experienced in recent decades, noncommunicable diseases, including CVDs, have dramatically increased in prevalence, emphasizing the urgency to accelerate population and high-risk approaches for CVD prevention.

In this special issue of *JACC: Asia* on Cardiovascular Disease Prevention, we feature 5 state-of-the-art reviews and 12 original research contributions, as well as a brief report and viewpoint, that comprise a broad portfolio of topics involving CVD epidemiology and prevention. Our reviews begin with a discussion by Patel et al^3^ of drivers of atherosclerotic CVD in South Asians, where emerging biomarkers, imaging techniques, and genomics-based approaches are noted to be promising for improving risk assessment and guiding treatment, but their utility, especially the cost-effectiveness in South Asians, needs further validation. Following this is a review by Ching et al^4^ on race and ethnicity in CVD prediction for multiethnic populations, where it is noted that in Asia, the path toward race-neutral risk prediction requires strengthening the data infrastructure and increasing participation in clinical trials. This is followed by a paper on the role of coronary calcium scoring and computed tomography angiography in assessing CVD risks in South Asians by Kalra et al,^5^ indicating that coronary calcium scoring may miss prevalent noncalcified and high-risk plaque in South Asians and that computed tomography angiography may be helpful to enable a more comprehensive assessment of plaque burden and composition in such patients. In a review on cardiovascular health promotion and disease prevention in China by Li et al,^6^ the need to address suboptimal adherence to healthy lifestyles and treatment strategies in primary and secondary prevention measures, with collaborative efforts involving individuals taking primary responsibility for their own health, is discussed. The final review by Dewi et al^7^ discusses the cardiovascular effects of electromagnetic fields, and given the rising adoption of electric vehicles worldwide, there is a need for collaboration to monitor electromagnetic field exposure levels and update safety guidelines because the long-term effects remain uncertain; the authors note the need for longitudinal studies assessing chronic exposure to low-frequency electromagnetic fields.

We also wish to highlight several original contributions in this issue. This includes a design paper on the recently started MOSAAIC (Multi-Ethnic Observational Study in American Asian and Pacific Islander Communities) led by Ahn et al^8^ This study is designed to improve the understanding of disparities in CVD and other conditions affecting Asian American and Native Hawaiian and Pacific Islander groups, and will enroll more than 11,000 adults among 5 centers in the United States. In a report by Zhang et al^9^ using data from the Global Burden of Disease Study between 1990 and 2021, ischemic heart disease and stroke together explained more than 80% of CVD deaths in Asia with elevated systolic blood pressure as the leading risk factor and particulate matter pollution being a leading contributor to CVD burden. Ali et al^10^ present an interesting holistic lifestyle framework to evaluate CVD disparities among older Thai adults showing the relation of unhealthy behaviors and poor stress management with prevalent CVD, stressing the need for holistic attention to behavioral risks and the importance of lifestyle medicine to support recovery and healthy aging in CVD patients. Among a large national cohort of Indian patients with coronary artery disease, Puri et al^11^ discuss important gaps in lipid treatment where despite high-intensity statin use, only one-quarter of patients achieved target low-density lipoprotein cholesterol levels, calling for urgent action to improve treatment efforts in this high-risk population. Finally, among original research contributions, an interesting paper by Iwasaki et al^12^ presents in a large Japanese cohort the relation of constipation with incident CVD. The issue then concludes with a brief report on CVD and life expectancy in chronic kidney disease patients and a viewpoint on women’s cardiovascular disease prevention in Asia.

Although this special issue of *JACC: Asia* highlights the work from investigators throughout the Asia-Pacific region, it is hoped that the future will bring many more important contributions that will continue to showcase the fine work of many scholars to advance CVD epidemiology and prevention efforts in the Asia-Pacific region. Such continued efforts will hopefully further motivate improved implementation of primordial, primary, and secondary prevention efforts aimed to reduce the CVD burden in the region.

## Funding Support and Author Disclosures

The authors have reported that they have no relationships relevant to the contents of this paper to disclose.
